# Nanoparticles as New Antifungals in the Prevention of Bovine Mycotic *Mastitis* Caused by *Candida* spp. and *Diutina* spp.—In Vitro Studies

**DOI:** 10.3390/molecules30102086

**Published:** 2025-05-08

**Authors:** Magdalena Kot, Agata Lange, Weronika Jabłońska, Aleksandra Kalińska, Barbara Nasiłowska, Wojciech Skrzeczanowski, Marcin Gołębiewski

**Affiliations:** 1 Animal Breeding Department, Warsaw University of Life Sciences, 02-786 Warsaw, Poland; s207303@sggw.edu.pl (W.J.); aleksandra_kalinska@sggw.edu.pl (A.K.); marcin_golebiewski@sggw.edu.pl (M.G.); 2 Department of Nanobiotechnology, Warsaw University of Life Sciences, 02-786 Warsaw, Poland; agata_lange1@sggw.edu.pl; 3 Institute of Optoelectronics, Military University of Technology, gen. S. Kaliskiego 2, 00-908 Warsaw, Poland; barbara.nasilowska@wat.edu.pl (B.N.); wojciech.skrzeczanowski@wat.edu.pl (W.S.)

**Keywords:** *mastitis*, *Candida* spp., *Diutina* spp., nanoparticles, fungi, dairy cattle, mycotics

## Abstract

Bacterial infections are the primary cause of *mastitis* in dairy cattle. Fungal *mastitis* occurs in 1–12% of cases. Antibiotic therapy, the standard treatment for *mastitis*, has led to antibiotic-resistant bacteria, reducing treatment efficacy and increasing fungal *mastitis* occurrence. Antibiotics lack biocidal effects on fungi, which often exhibit resistance to antifungal agents. This study evaluated the antifungal properties of nanoparticles (NPs) against *Candida albicans*, *Candida glabrata*, *Candida parapsilosis*, *Diutina rugosa* var. *rugosa*, *Diutina catenulata*, and *Diutina rugosa*. Tested NPs included gold (AuNPs), silver (AgNPs), copper (CuNPs), iron with hydrophilic carbon coating (FeCNPs) (1.56–25 mg/L), and platinum (PtNPs) (0.625–10 mg/L), along with their complexes. Minimum inhibitory concentration (MIC) and minimum fungicidal concentration (MFC) at 0.75–25 mg/L for AuNPs, AgNPs, CuNPs, and FeCNPs and 0.313–10 mg/L for PtNPs, as well as fungal sensitivity to standard antifungals, were determined. Each strain showed different sensitivities depending on the NPs used and their concentrations. *C. glabrata* was the most resistant to nanoparticles, while *D. catenulata* was the most susceptible. PtNPs and FeCNPs showed no or weak biocidal properties. Some mycotic-resistant strains were sensitive to nanoparticles. This study indicates a high in vitro antifungal potential for the application of nanoparticles, especially AgCuNPs, as a new effective non-antibiotic agent for the prevention and control of mycotic *mastitis* in dairy cattle.

## 1. Introduction

*Mastitis*, also known as udder inflammation, occurs in dairy cattle and is a multi-etiological complex disease, most often associated with an etiological agent such as bacteria. The most common contributors to *mastitis* are bacteria, such as *Escherichia coli*, *Staphylococcus aureus*, *Streptococcus uberis*, *Streptococcus dysgalactiae*, *Streptococcus agalactiae*, and *Staphylococcus epidermidis* [1]. Although *mastitis* is most often mentioned in the context of bacterial infections, it is not limited to this etiological agent. It is a fact that bacteria are most often isolated from infected quarters of the udder; however, *mastitis* can also be induced by fungi, but at a much lower frequency. These include species of yeasts and molds. The most commonly isolated mastitis-inducing yeast is *Candida* spp.: *C. kefir*, *C. rugosa*, *C. krusei* [2], *C. glabrata*, *C. tropicalis*, *C. pseudotropicalis*, and *C. parapsilosis* [3]. Another decidedly less frequently isolated *mastitis*-inducing species is *Diutina* spp., e.g., *D. rugosa* [4]. In recent years, the occurrence of protothecal *mastitis* has also been increasingly reported; this type of *mastitis* is caused by a pathogenic alga of the genus *Prototheca*, which is particularly resistant to conventional treatment [5]. Mixed-type infections are also common. The fungi that cause *mastitis* are classified as environmental pathogens. Their occurrence is significantly influenced by the environment. Although fungal *mastitis* is much less common than bacterial *mastitis*, leading to its effects being underestimated, it should be noted that it still poses a serious threat to animal health. Fungal species isolated from milk from cows with confirmed *mastitis* exhibit resistance to antifungal drugs similar to that exhibited by bacterial strains to antibiotics, which means that strains targeted by biocides have varying susceptibility. In addition, in veterinary practice, the identification of the type of fungi is not performed due to their rarer occurrence. At the same time, bacteria are more frequently the main etiological factor in the development of *mastitis*, leading to the adoption of a treatment strategy using antibiotics, which in turn leads to an increase in the incidence of fungal *mastitis* [6]. Unfortunately, it is not only that antibiotic treatments might be ineffective due to increasing antibiotic resistance among bacterial strains but also that some antibiotics can act as nutrients for fungal strains. Examples of such types of antibiotics that are in popular use are tetracycline and penicillin—these contain nitrogen, which fungi treat as a source of energy to support their growth [7]. Therefore, antibiotic-based *mastitis* treatment does not give producers confidence that it will provide a positive biocidal effect against the most common etiological agents, and at the same time, the treatment may intensify the problem of fungal *mastitis*. It should be remembered that fungal strains, like bacteria, also have characteristic mechanisms for defense, including the ability to form biofilm, which makes them more resistant to unfavorable conditions in the external environment and, therefore, more resistant to applied antifungal agents [8].

The purpose of this study was to determine the basic physicochemical properties of the utilized nanoparticles (AuNPs, AgNPs, CuNPs, FeCNPs—iron NPs coated with carbon—and PtNPs) and nanoparticle complexes that showed the strongest biocidal activity, as well as to determine their antifungal properties against one of the most commonly isolated—*Candida* spp. (*C. albicans*, *C. glabrata*, and *C. parapsilosis*)—and some of the less commonly isolated species, *D. rugosa* var. *rugosa*, *D. catenulata*, and *D. rugosa*, isolated from the milk of cows diagnosed with subclinical *mastitis*. In addition, the sensitivity of the isolated microorganisms to available antifungal substances was determined (amphotericin B 20 g/L, econazole 10 g/L, flucytosine 1 g/L, fluconazole 10 g/L, fluconazole 25 g/L, ketoconazole 10 g/L, clotrimazole 10 g/L, and nystatin 100 g/L) using the disc diffusion method. This study also determined the minimum inhibitory concentration (MIC) and minimum fungicidal concentration (MFC) of AgNPs, AuNPs, CuNPs, PtNPs, FeCNPs—iron NPs coated with carbon—and their complexes at concentrations of 0.75, 1.56, 3.125, 6.25, 12.5, and 25 mg/L; 0.313, 0.625, 1.25, 2.5, 5, and 10 mg/L were used for PtNPs. This research provides insight into and perspective on the potential application of nanoparticles in practice for the prevention of *mastitis*. This is the first publication in the literature that builds on such a wide variety and comprehensiveness of the conducted analyses, targeting pathogens that have so far been underestimated. The results fill the gaps in previous studies and make an incredibly valuable contribution to the development of research on the control of fungal *mastitis* in dairy cows using NPs. This is an evaluation of a potential prevention method for herds affected by fungal *mastitis* based on nanoparticles, while allowing effective prophylaxis in healthy udders; however, in vivo studies are also required.

## 2. Results

### 2.1. The Distribution and Occurrence of Isolated Pathogens from Quarter Milk Samples

The preliminary results of tests to identify fungal species for further study are presented in Table 1, which shows the quantitative and qualitative distribution of isolated microorganisms. Out of 67 milk samples, the majority either did not contain microorganisms (32.84%) or had bacterial strains isolated from them (35.82%). In contrast, 20.89% of samples contained fungal strains, which were then subjected to identification.

### 2.2. Identification of the Isolated Fungi

In the conducted studies, the following fungi were isolated: *C. albicans*, *C. glabrata*, *C. parapsilosis*, *D. rugosa* var. *rugosa*, *D. catenulata*, and *D. rugosa*. The colonies that were grown were identified using a MALDI-TOF MS apparatus (Bruker, Poznań, Poland). Fungal identification was based on mass spectrometry, and the results are shown in Table 2.

### 2.3. Nanoparticle Morphology

**Figure 1:**
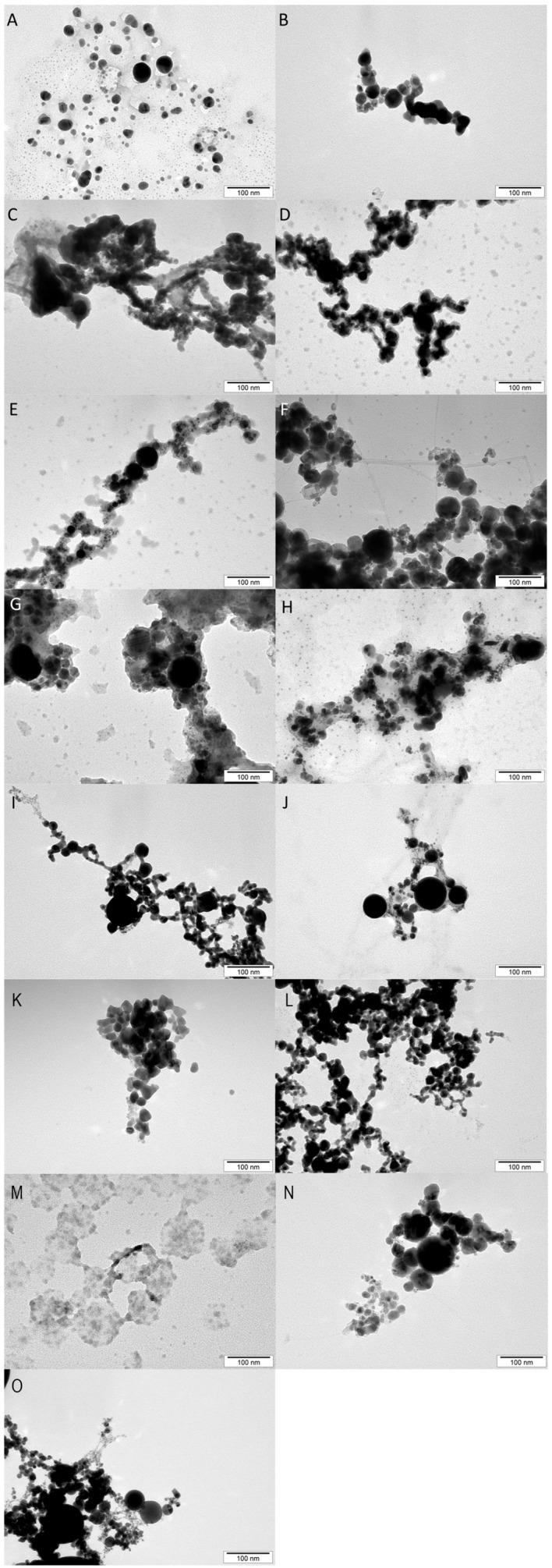
Nanoparticle images taken using a transmission electron microscope (TEM). (**A**)—AgAu, (**B**)—AuFeC, (**C**)—CuAg, (**D**)—CuAu, (**E**)—CuPt, (**F**)—CuFeC, (**G**)—FeCAg, (**H**)—PtAg, (**I**)—PtAu, (**J**)—PtFeC, (**K**)—Ag, (**L**)—Au, (**M**)—Cu, (**N**)—FeC (iron NPs coated with carbon), (**O**)—Pt.

The morphology of the nanoparticles is presented in Figure 1. The images of the nanoparticles were taken with a transmission electron microscope (TEM) and illustrate the morphology of the metal nanoparticles used in the experiment. All of the tested nanoparticles show a spherical morphology, which follows the morphological standard for nanoparticles, indicating that they have a standardized quality. Only in the case of CuNPs was an atypical appearance observed, which suggests the presence of impurities, probably due to the nanoparticle production process (this is especially evident in the images in Figure 1C,M).

### 2.4. Physicochemical Properties Analysis of the Nanoparticles: Hydrodynamic Diameter (nm) and Zeta Potential (mV)

**Figure 2:**
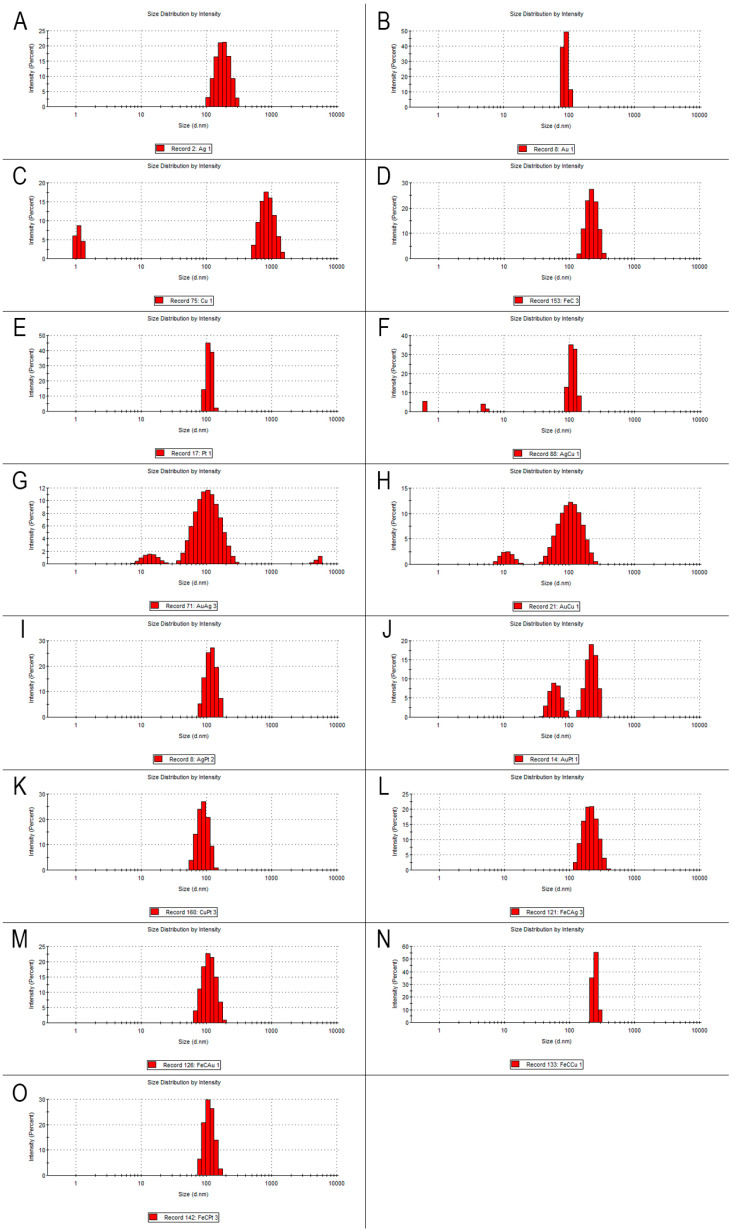
The hydrodynamic diameter of the nanoparticles. (**A**)—Ag, (**B**)—Au, (**C**)—Cu, (**D**)—FeC (iron NPs coated with carbon), (**E**)—Pt, (**F**)—AgCu, (**G**)—AuAg, (**H**)—AuCu, (**I**)—AgPt, (**J**)—AuPt, (**K**)—CuPt, (**L**)—FeCAg, (**M**)—FeCAu, (**N**)—FeCCu, (**O**)—FeCPt.

Figure 2 shows the basic physicochemical properties of the nanoparticles and their complexes used in this experiment, namely the hydrodynamic diameter, which determines the size of the formed agglomerate. Table 3 presents the detailed numerical values of the physicochemical parameters.

The size distribution of the nanoparticles shown in Figure 2 indicates that three separate size assemblages were present. Two different size assemblages were observed for CuNPs (Figure 2C); the smallest assemblage of nanoparticles measured about 1 nm, while the largest oscillated around 1000 nm. There were also two size assemblages for AuCuNPs (Figure 2H), but here, the smallest assemblage was 7–20 nm and the largest was 40–300 nm, while most nanoparticles were around 100 nm. Double-size assemblages also occurred for the AuPtNP complex, whose lowest values were 40–100 nm and whose highest values were 200–300 nm. Triple-size assemblages occurred for the AgCuNP (Figure 2F) and AuAgNP (Figure 2G) complexes. In the first case, the nanoparticles in the size assemblages were less than 1 nm, less than 10 nm, and about 100 nm, while the AuAgNP complex had values above 10 nm, with the larger assemblages being about 100 nm; this complex also had the largest of all size assemblages, at 4000–6000 nm. In the case of the other nanoparticles and complexes, most values were around 100 nm, but some values reached as high as 500 nm.

The zeta potential of the nanoparticles and their complexes was determined using dynamic laser scattering under electrophoretic conditions, the results of which are shown in Supplementary Figure S1. The zeta potential for some nanoparticles took various values, but all of them were negative. The greatest variation in the measurements of this parameter was observed for the AgCuNP complex (Supplementary Figure S1F), and slightly less for AuCuNPs (Supplementary Figure S1H), AuPtNPs (Supplementary Figure S1J), and FeCCuNPs (Supplementary Figure S1N). Small variations between measurements were observed for the other nanoparticles.

The largest hydrodynamic diameters for single nanoparticles were observed for FeCNPs (iron NPs coated with carbon) (746.9 nm) and CuNPs (652.1 nm), while the complex with the highest average hydrodynamic size was the CuFeCNP complex, reaching 743.7 nm. The smallest diameter was observed for AgNPs (201.6 nm), while the smallest complex was AgPtNPs, reaching 106.1 nm.

Negative zeta potential values indicate the type of surface charge on the nanoparticles. Among the single nanoparticles, CuNPs had the least negative zeta potential (−3.5 mV), indicating low colloidal stability, but when considering the morphology of the nanoparticles used in this study, this value indicates physicochemical abnormalities, most likely related to the formation process. The AgNPs had the most negative value among the single nanoparticles (−35.2 mV), while among the complexes, AgPtNPs had the most negative value (−38.1 mV). In general, the zeta potential values indicated the samples had moderate to low stability. The highest PdI value was observed for FeC (0.8 ± 0.8), while the lowest was observed for AgFeC (0.3 ± 0.1). A PdI value approaching 1 indicates high polydispersity and high particle size variation.

### 2.5. Laser Emission Spectroscopy Studies of the Tested Nanoparticles

**Figure 3:**
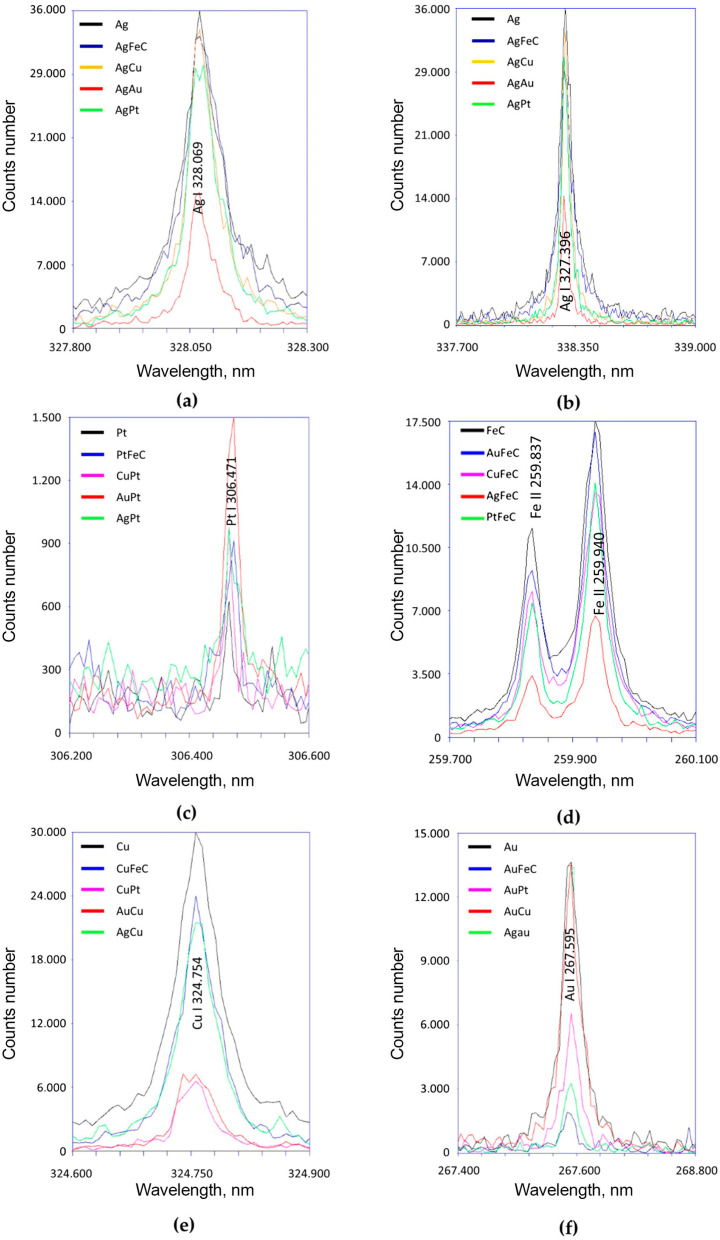
Laser emission spectroscopy studies of selected NPs using different spectral lengths. Where the (**a**–**f**) diagram shows measurements for the studied nanoparticles, for different lengths of the spectra.

Laser emission spectroscopy allowed the confirmation of the presence of the tested NPs; Ag (328.069 and 338.289 nm) (Figure 3a,b), Pt (306.471 nm) (Figure 3c), Fe (259.837 and 259.940 nm) (Figure 3d), Cu (324.754 nm) (Figure 3e), and Au (267.595 nm) (Figure 3f) were contained in all dispersed aqueous suspensions tested. The results showed that the highest peak intensity occurred in suspensions containing single nanoparticles, the exception being PtNPs. These differences may be due to the uneven distribution of the dried nanoparticles. It is worth noting the low intensity of Pt peaks, which indicates the low content of these nanoparticles in the volume of the liquid tested.

### 2.6. Elemental Composition Tests of Nanoparticles (EDX—Energy-Dispersive X-Ray)

The results of the EDX analysis with standard deviations are presented in Table 4. The study confirmed the presence of NPs. The number of NPs varied depending on the type of NPs, and the differences could be due to the formation of agglomerates.

### 2.7. Fungicidal Properties of the Tested Nanoparticles

The survival rate results of the isolated fungal strains are presented in Figure 4, Figure 5, Figure 6, Figure 7, Figure 8, Figure 9, Figure 10 and Figure 11.

#### 2.7.1. Silver Nanoparticles

**Figure 4:**
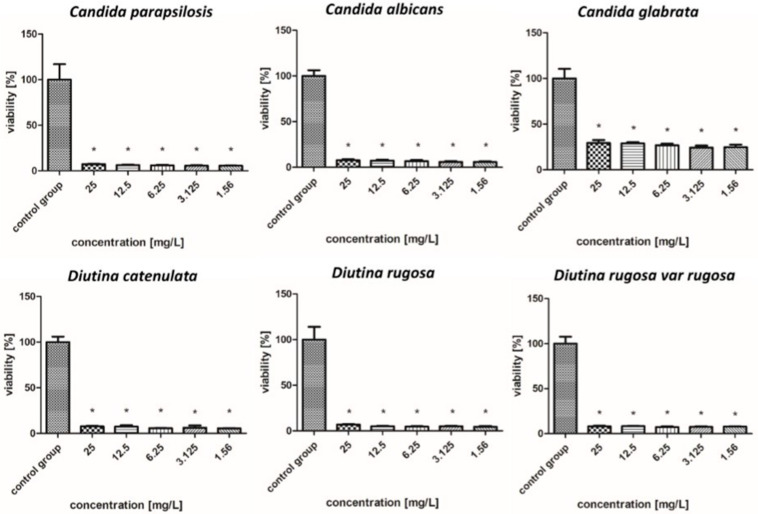
Viability (%) of the fungal strains exposed to silver nanoparticles. The results are presented as the mean ± standard deviation, and asterisks (*) show statistically significant differences (*p* ≤ 0.05) compared with the control group.

As shown in the results presented in Figure 4, AgNPs showed similar levels of biocidal activity regardless of the applied concentration; however, the activity varied depending on the fungal strain. The applied AgNPs showed the strongest biocidal properties against *D. rugosa* and *C. albicans*, whose survival rate in both cases for each applied concentration was 7–5%. Similar survival rates were also observed for *D. catenulata* and *C. parapsilosis*; the survival rate for the highest concentration of 25 mg/L was 8%, while for the lowest concentration, it was 6%. In the case of *C. glabrata*, the survival rate for the lowest applied concentration was 22%.

#### 2.7.2. Gold Nanoparticles

**Figure 5:**
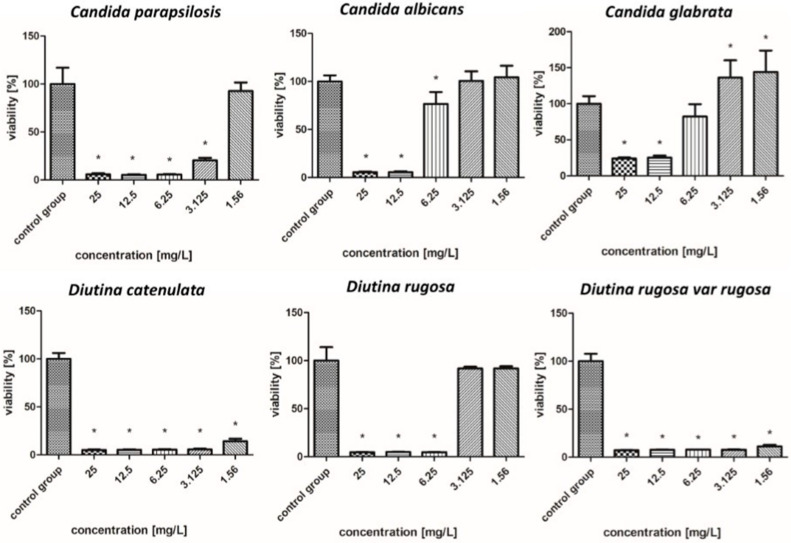
Viability (%) of the fungal strains exposed to gold nanoparticles. The results are presented as the mean ± standard deviation, and asterisks (*) show statistically significant differences (*p* ≤ 0.05) compared with the control group.

Figure 5 shows that in the case of AuNPs, pathogen survival increased as the concentration decreased. These nanoparticles showed weaker biocidal properties than AgNPs. The applied nanoparticles showed the weakest antifungal properties against the *C. albicans* and *C. glabrata*, whose survival rate increased from 5–22% for a concentration of 25–6.25 mg/L to 104% for a concentration of 3.125–1.56 mg/L. In the case of *C. parapsilosis*, across all concentrations, the survival rates were 6–24%, but for the lowest concentration of 1.56 mg/L, there was an increased survival rate, reaching more than 100%. *D. catenulata* and *D. rugosa* var. *rugosa* were the most sensitive pathogens to AuNPs, where the survival rate for the highest concentration was 5–7%, and that for the lowest concentration, 1.56 mg/L, was 15–11%.

#### 2.7.3. Copper Nanoparticles

**Figure 6:**
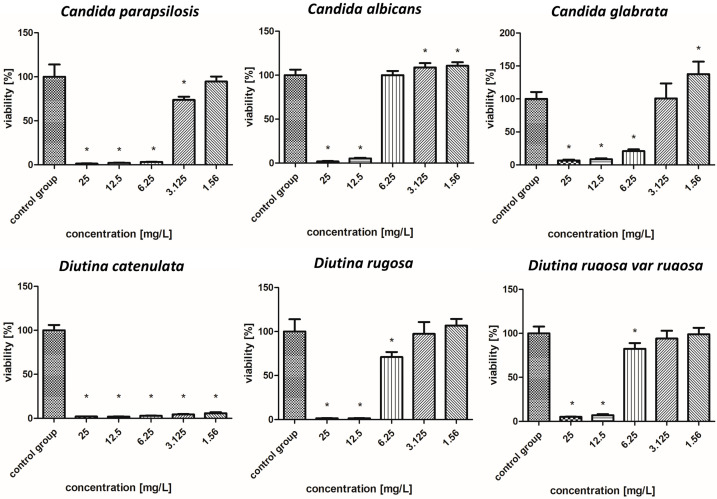
Viability (%) of the fungal strains exposed to copper nanoparticles. The results are presented as the mean ± standard deviation, and asterisks (*) show statistically significant differences (*p* ≤ 0.05) compared with the control group.

As shown in Figure 6, overall, CuNPs showed improved antifungal properties compared to AuNPs but worse antifungal properties compared to AgNPs. *D. catenulata* was the most susceptible pathogen to CuNPs; at concentrations of 6.25–25 mg/L, it showed very weak survival rates of about 2%, whereas at concentrations of 1.56 and 3.125 mg/L, the values increased, but not significantly, with a survival rate of only 4–5%. In the case of *C. albicans*, *C. glabrata*, *D. rugosa*, and *D. rugosa* var. *rugosa*, high concentrations, i.e., 12.5 and 25 mg/L, reduced the survival rate to 1–7%, while with decreasing concentrations, there was an increase in survival rate, reaching more than 100% in some cases. In contrast, the survival rate of strains such as *C. parapsilosis* for concentrations of 6.25–25 mg/L ranged from 3% to 8%.

#### 2.7.4. Iron Nanoparticles Coated with Carbon

**Figure 7:**
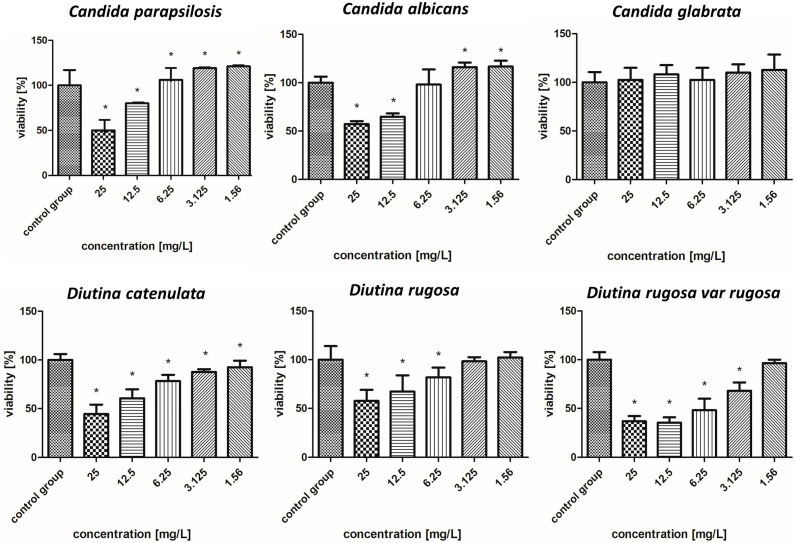
Viability (%) of the fungal strains exposed to iron nanoparticles. The results are presented as the mean ± standard deviation, and asterisks (*) show statistically significant differences (*p* ≤ 0.05) compared with the control group.

According to Figure 7, FeCNPs—iron NPs coated with carbon—achieved worse effects than CuNPs. In the case of *C. albicans*, *D. catenulata*, *D. rugosa*, and *D. rugosa* var. *rugosa*, when the highest concentrations were used, the survival rates of these pathogens oscillated around 37% to 61%, which increased as the concentration decreased. The most resistant pathogen to the FeCNPs was *C. glabrata*, for which even the highest concentrations showed low or no fungicidal properties, and the most susceptible was *D. rugosa* var. *rugosa*.

#### 2.7.5. Platinum Nanoparticles

**Figure 8:**
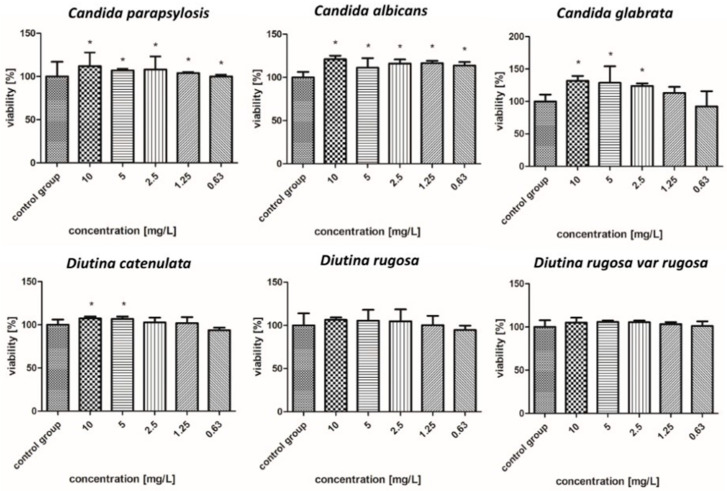
Viability (%) of the fungal strains exposed to platinum nanoparticles. The results are presented as the mean ± standard deviation, and asterisks (*) show statistically significant differences (*p* ≤ 0.05) compared with the control group.

As presented in Figure 8, by far the worst results of all the tested nanoparticles regarding fungicidal activity were observed for PtNPs. For all other strains, PtNPs showed poor or no decrease in survival, even for the highest concentrations. Due to the weak antifungal activity of iron and platinum nanoparticles, they were not considered for further study of their effects in nanocomplexes.

#### 2.7.6. Silver–Gold Nanocomplex

**Figure 9:**
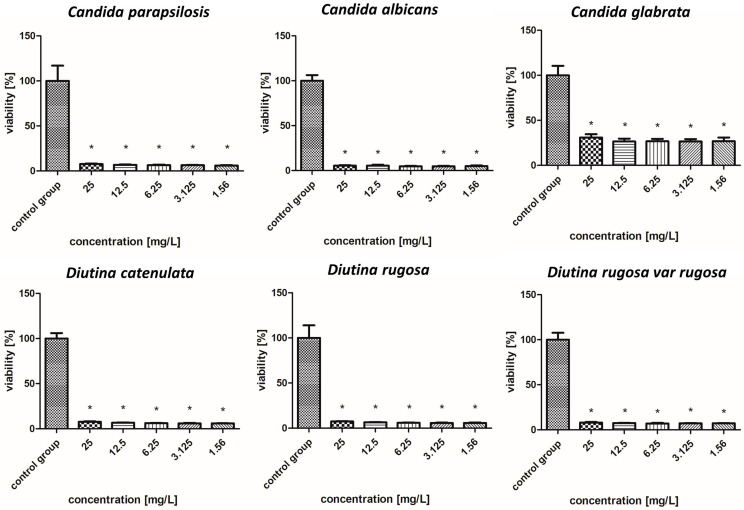
Viability (%) of the fungal strains exposed to silver–gold nanocomplex. The results are presented as the mean ± standard deviation, and asterisks (*) show statistically significant differences (*p* ≤ 0.05) compared with the control group.

Figure 9 illustrates that the nanoparticle complexes showed synergistic effects and achieved stronger fungicidal properties than single nanoparticles, but to varying degrees. The AgAuNP complex showed a similar tendency to AgNPs based on the fact that there were no rapid increases or decreases in the level of a given strain in relation to the concentration of nanoparticles. Strains such as *C. albicans*, *C. parapsilosis*, *D. catenulata*, *D. rugosa*, and *D. rugosa* var. *rugosa* were most sensitive to the complex, with survival rates for each concentration ranging from 5 to 8%. In the case of *C. glabrata*, the applied complex showed lower efficacy, but nevertheless, the survival rate was 25–35%, depending on the concentration.

#### 2.7.7. Silver–Copper Nanocomplex

**Figure 10:**
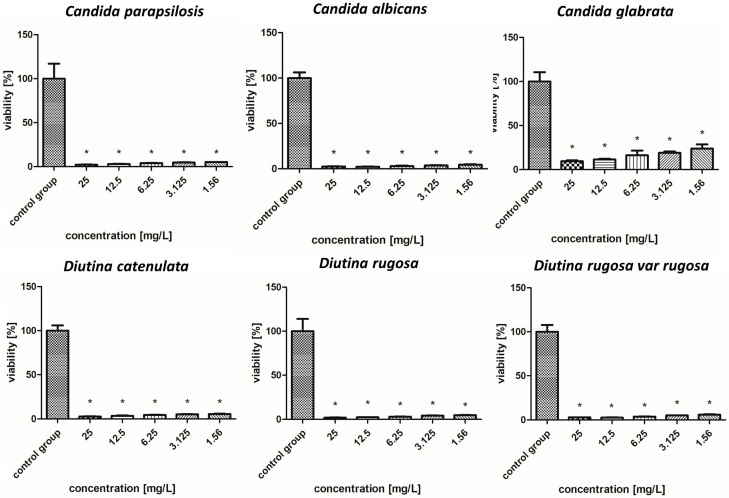
Viability (%) of the fungal strains exposed to silver–copper nanocomplex. The results are presented as the mean ± standard deviation, and asterisks (*) show statistically significant differences (*p* ≤ 0.05) compared with the control group.

Regarding Figure 10, the complex that showed the strongest fungicidal activity among all the tested individual nanoparticles and nanocomplexes was AgCuNPs. For strains of *C. albicans*, *C. parapsilosis*, *D. catenulata*, *D. rugosa*, and *D. rugosa* var. *rugosa*, the survival rate at the highest concentration was about 2%. Slightly weaker antifungal activity was observed for *C. glabrata*, whose survival rate was 10%.

#### 2.7.8. Gold–Copper Nanocomplex

**Figure 11:**
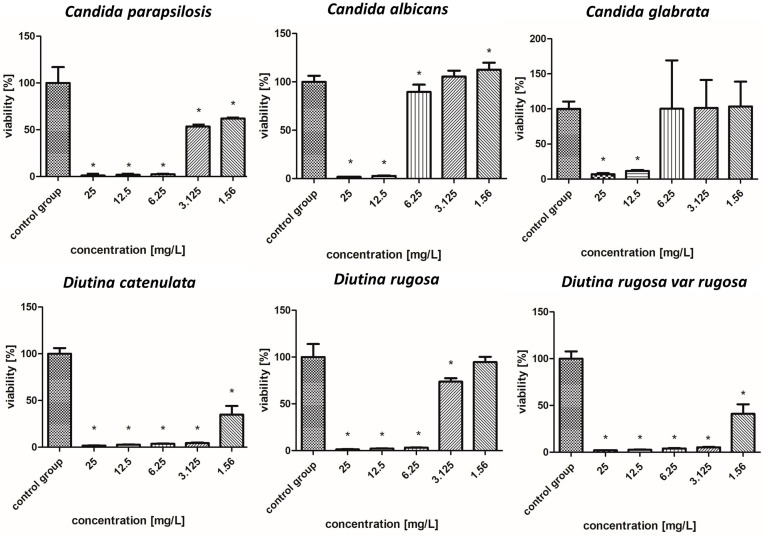
Viability (%) of the fungal strains exposed to gold–copper nanocomplex. The results are presented as the mean ± standard deviation, and asterisks (*) show statistically significant differences (*p* ≤ 0.05) compared with the control group.

As indicated in Figure 11, the AuCuNP complex demonstrated the weakest fungicidal properties among the complexes, but its effect was nevertheless strong. The most sensitive pathogens to this complex were *D. catenulata* and *D. rugosa* var. *rugosa*, in which the highest concentration reduced the survival rate to 1–6%; however, an increase in survival rate at the lowest concentration was also observed (20–41%). Concentrations of 12.5–25 mg/L showed the strongest fungicidal properties against *C. albicans* and *C. glabrata*, reducing their survival to 1–11%, but at lower concentrations (1.56–6.25 mg/L), their survival increased rapidly. Strong biocidal properties were also observed in the case of *C. parapsilosis* and *D. rugosa*, in which, up to a concentration of 6.25 mg/L, the survival rate oscillated between 1 and 10%.

### 2.8. Minimum Inhibitory Concentration (MIC) and Minimum Fungicidal Concentration (MFC) Results After Incubation with Selected NPs

Based on the results presented in Table 5, it is possible to provide a detailed description of the effects of nanoparticles on particular strains of fungi. The results in the table represent the MIC and MFC after NP treatment.

Table 5 shows the pathogens for which MIC and MFC were obtained. If no MIC or MFC was obtained for a particular nanoparticle, the nanoparticle’s data were excluded. For strains for which one of the measurements could not be determined, “-” is marked in the table.

### 2.9. Antifungal Disc Results

The sensitivities of the isolated microorganisms to available mycotic substances using the disc diffusion method are presented in Table 6.

The largest growth inhibition zone (40 mm) was obtained for *C. parapsilosis* after KCA10 treatment, while after CTM10 treatment, the zone of inhibition was 34 mm. The *C. glabrata* strain, after using flucytosine 1 µg (FY1), had a growth inhibition zone with a radius of 32 mm. The KCA10 formulation was particularly effective against the *D. catenulata* and *D. rugosa* strains, reaching zones of inhibition of 30 mm for both strains. In contrast, the worst-performing agent was fluconazole 10 µg (FCN10) for the strains *C. parapsilosis*, *D. rugosa*, and *D. catenulata*, with values of 0 mm. *D. rugosa* var. *rugosa* with AMB20 applied reached a zone size of 6 mm, as did *C. parapsilosis* with the same agent. The AMB20 formulation also showed high efficacy with *C. parapsilosis*, *C. albicans*, *C. glabrata*, *D. catenulata*, and *D. rugosa*, resulting in inhibition zones of 10 mm for each of these strains. It is worth pointing out that the AMB20 formulation demonstrated different efficacies depending on the fungal strain. For *C. parapsilosis* and *D. rugosa* var. *rugosa*, the zones of inhibition were 8 mm, 6 mm, and 6 mm, respectively. In contrast, the highest values, 10 mm each, were obtained for *C. albicans*, *C. glabrata*, *D. catenulata*, and *D. rugosa*. Growth inhibition zones of 4 mm were obtained for the *C. glabrata* strain using CTM10 and 8 mm using FCN10.

## 3. Discussion

There are limited reports in the available literature of studies on the effects of NPs on fungal strains isolated from cows diagnosed with *mastitis*. However, among the available reports, a similar study was conducted by Zhang et al. [9]. In this study, silver nanoparticles (AgNPs) were shown to have potent antifungal activity against various fungal strains, including *C. albicans* and *C. glabrata*. In a study by Wierzbicki et al. [10], *C. albicans* was highly resistant to nanoparticles, but *C. parapsilosis* was sensitive to AuNPs. In our study, *C. parapsilosis* was susceptible to AuNPs, especially at concentrations higher than 6.25 mg/L. While a study by Wierzbicki et al. showed that the survival rate of *C. glabrata* after treatment with AuNPs was reduced by 62%, in our research, the survival rate was reduced by as much as 78% at the highest concentration of 25 mg/L. In their case, 25 mg/L CuAuNPs induced an 80% reduction in the pathogen’s colonies, while in our case, growth was inhibited by 93%. *D. rugosa* also ranks among rare fungi responsible for *mastitis* in cattle. *D. rugosa* infections are new to the veterinary literature. Kim et al. [11] determined the sensitivity of fungi to AgNPs using a broth microculture method. Sensitivity was tested for strains such as *Candida* and *Trichophyton mentagrophytes*, with the nanoparticles showing antifungal activity against both genera, which is consistent with our study, in which all three *Candida* strains showed high sensitivity to AgNPs. Khezerlou et al. [12] conducted a study on AuNPs, which showed efficacy against *C. albicans*. In our study, AuNPs were effective against most of the strains tested only at higher concentrations. A study by Yehia and Ali [13] showed that iron nanoparticles (FeNPs) generated antifungal activity against *Candida* spp. Compared to the drug AMB20, which they also used, the FeNPs were more effective in inhibiting the growth of *C. albicans* and *C. parapsilosis*. As reported by Osama et al. [14], AgNPs, CuNPs, and their complexes are effective in reducing pathogen survival in *mastitis* cases. The high effectiveness of complexes of these nanoparticles was proven in this study, where the average survival rate for all concentrations of AgCuNPs for all strains was only 11%. Fan et al. [15] reported that AgNPs are more effective against fungi and yeast compared to CuNPs; however, the results obtained in our study differ from the study by Fan et al.

In the case of *mastitis* caused by *D. rugosa*, according to Bhordia et al. [16], the pathogen is sensitive to some commercial drugs, such as FCN. It has been proven repeatedly that *D. rugosa* is also resistant to AMB20. Our study also confirms this fact concerning resistance to AMB20 and sensitivity to FCN25. The results of a study by Paredes et al. [17] showed that, against the mentioned strain, AMB20 showed an average minimum inhibitory concentration (MIC) value of 0.66 mg/L, while the MIC for FCN averaged 0.81 mg/L. A determination of the sensitivity of antifungal drugs commonly found in human and veterinary medicine was carried out by Stavrou et al. [18]. They further described that AMB is an excellent antifungal therapy against most known strains. On the other hand, a paper by Al-abedi and Khalil [19] showed that 50% of *C. parapsilosis* strains were sensitive to FCN, 45% were sensitive to KCA, 25% were sensitive to NY, and 75% were sensitive to AMB20. Another study conducted by Hasanin et al. [20] involving *C. albicans*, *C. tropicalis*, *P. kudriavzevii*, and *C. glabrata* showed that all the *Candida* isolates used were sensitive to KCA, with zones of inhibition ranging from 24.7 mm to 34.3 mm. In our case, the zone of inhibition for one of the strains, *C. glabrata*, was lower (14 mm in diameter), indicating that the strain was resistant to KCA. However, after applying other antifungals to *C. glabrata*, we proved that this strain is sensitive to FY1, as the zone of inhibition here was 32 mm (diameter). Our results confirm the results obtained by Hasanin et al. for *C. glabrata*, in which this strain showed low survival only at higher concentrations of AgNPs. Hasanin et al. also calculated MIC values for AgNPs. Against *C. albicans*, they obtained MIC values of 0.7 μg/mL and 1 μg/mL. Against *C. glabrata*, the values were 2.7 μg/mL and 4 μg/mL. Ludwig et al. [21] also conducted MIC tests on AgNPs against *C. albicans*; the lowest inhibitory concentration was 0.21 mg/L, while against *C. parapsilosis*, it was 1.69 mg/L. They calculated MIC values for commercial antifungal drugs as well, which we tested using the disc diffusion method. The results of the study by Ludwig et al. are consistent with those obtained in our experiments, which were conducted on 96-well plates, as shown by the efficacy of AgNPs against various *Candida* strains. Ludwig et al. reported that, against their strains, KCA and FY proved to be the most effective treatment among the other mycotics tested. This agrees with our disc test, in which the largest diameter for the zone of inhibition was obtained for KCA (22 mm). FY, on the other hand, showed worse efficacy in our study. Another paper by Montoya et al. [22] also focused on the determination of MIC values for commercial mycotics conducted against *D. rugosa*. The MIC determined for AMB20 was ≤1 µg/mL, indicating that this formulation had high efficacy against *D. rugosa.* FCN (MIC ≤ 2 µg/mL) was also effective; however, in our study, FCN10 was not effective (diameter, 0 mm). Sukumar [23] conducted a study on *C. parapsilosis*, proving that these strains were sensitive specifically to AMB (100 U), CTM10, NY100, and KCA (100 mg). However, only 30% of the strains tested were sensitive to FCN (10 mg).

Pointing out the potential of using nanoparticles in practice, it is also worth highlighting their effect on cells. A study by Chen et al. [24] conducted toxicity tests of AgNPs against red blood cells. The study showed that the hemolysis of these cells varied depending on the NP dose and particle size. Smaller NPs, i.e., reaching 15 nm, showed higher hemolytic capacity than larger ones reaching 50 and 100 nm. A similar conclusion was drawn by Kim et al. [25], whose study pointed out that larger NP sizes (100 nm versus 30 nm) showed less toxicity to blood cells. Similar studies were conducted on chicken embryo blood cells [26]. AgNPs at 2 mg/L in these studies showed a negative effect of these nanoparticles on the morphology of red blood cells, leading to anisocytosis and an increase in the number of immature erythrocytes. Still, they did not lead to hemolysis of these cells. Similar results were obtained in a study by Aseichev et al. [27], in which the hemolysis of nanoparticles decreased with increasing nanoparticle size (5, 10, and 20 nm), but the tests conducted for Au concentrations of 20 μM did not cause hemolysis. Other results were obtained in a study by Purohit et al. [28], whose research showed the toxicity of AuNPs to human red blood cells, but the application of a coating of bovine serum albumin on the NPs caused them to exhibit much lower hemolysis. A study by Kote et al. [29] examining the effects of CuNPs on red blood cells indicated that low concentrations of these NPs (1 mg/mL) and a size of 22 nm showed no toxicity to these cells. Similar results were obtained by Abdulwahab et al. [30], who conducted an experiment on different concentrations of CuNPs: 200, 100, 50, 25, 12.5, and 6.25 mg/L. The study showed that red blood cell hemolysis for a concentration of 100 mg/L was less than 5%. However, it is worth highlighting that the survival rate of red blood cells varies depending not only on the size and concentration of NPs but also on the methods of their synthesis, which should be taken into account when considering the possibility of using NPs in practice.

A similar tendency is observed for other cells, for example, human mammary epithelial cells (BME-UV1) and human microvascular endothelial cells (HMEC), on which studies were conducted by Wierzbicki et al. [10], showing that among the Ag, Au, Cu, Fe, PtNPs and their complexes, only CuNPs and copper-containing complexes showed a reduction in the survival of the aforementioned cells at concentrations higher than 1 mg/L. Slight toxicity (10–15%) was also noted for the highest concentrations. Similar results were obtained by Radzikowski et al. [31] using BME-UV1 cells, which showed that concentrations of 0.5–5 mg/L of AuNPs showed no toxicity to these cells. Another study by Lange et al. [32] using HFFF2 lineage cells and 1 µg/mL Ag, Au, and Cu NPs showed no toxicity. The indicated reports suggest great potential for the use of nanoparticles in the prevention of fungal *mastitis*, but differences in the effects of individual NPs must be considered.

The mechanisms of action of nanoparticles differ, with different potential mechanisms of action in the prevention of *mastitis*. Silver nanoparticles slowly release silver ions. These ions are highly reactive and can interact with bacterial/yeast cell components, and they can bind to thiol groups in the pathogen’s proteins and enzymes, thereby interfering with essential cellular processes like energy production and replication. The literature indicates that AgNPs are able to enter the pathogen’s cytoplasm and interact with their DNA and ribosomes, which results in DNA condensation and, later, mutations. Ribosomes, the cellular machinery responsible for translating genetic information into functional proteins, are directly targeted by nanoparticles, leading to the disruption of protein synthesis. This interference halts the production of essential proteins required for bacterial growth and metabolism [33,34]. Based on the findings reported by Długosz et al. [35], it can be inferred that the silver–copper nanoparticle systems (Ag-Cu) utilized in the present study exhibit significant antimicrobial efficacy, consistent with the observations made for nanoparticles synthesized in deep eutectic solvents (DESs). Although not explicitly defined as a chemical “complex”, the Ag-Cu system is understood as a coexistent formulation comprising both silver (AgNPs) and copper nanoparticles (CuNPs) within a unified matrix. The incorporation of these metallic nanoparticles into DES-based systems has been shown to improve their physicochemical stability under variable environmental conditions while maintaining sustained bioactivity over time. Similarly, this was also proven in the work of Martín-Perales et al. [36], in which the combination of silver and copper in a single “system” showed significantly better antimicrobial efficacy than the individual metals used separately. The synergy of silver and copper nanoparticles is based on their complementary properties, where silver releases the Ag+ ions mentioned many times before, while copper also releases Cu^2+^ ions. The material used in Martín-Perales et al.’s Ag-Cu@ZIF-8 work was characterized by high porosity, which allows for efficient deposition and controlled release of metal ions, further aiding their long-term performance in the system. The bimetallic bonding of the commercial nanoparticles themselves, as in our study, also creates the conditions for the ion release demonstrated in the mentioned works. Such properties are particularly important in the context of increasing antimicrobial resistance, as the synergy between metals enhances effectiveness against infections, including *mastitis*.

Research on the application of nanoparticles directly to the udder is still in progress, and some commercially available products are already offered to breeders. Nevertheless, scientific data on the agents discussed in this article, among others, are still lacking in terms of safety. Furthermore, in vitro studies are still common. One paper, authored by Garibo Ruiz et al. [37], involves testing nanoparticles in vivo. The aim of the Garibo Ruiz et al. study was to evaluate the effects of Argovit-C, which contains AgNPs, on the susceptibility of *S. aureus* and *S. dysgalactiae* to antibiotics in cows suffering from *mastitis*. The results of the in vivo experiments indicated that Argovit-C AgNPs significantly improved the susceptibility of *S. aureus* and *S. dysgalactiae* to a wide range of antibiotics. These results mean that nanoparticles, which were the main target in our study as well, could be a promising tool in overcoming drug resistance in veterinary medicine. However, survival studies using fungal strains isolated from the affected udder are not practiced, and therefore, this represents a major research gap.

AgAu complexes showed varying efficiency values for various organisms. Such complexes form a complex “interface” structure, which can affect their catalytic activity. Reports indicate that the distribution of silver and copper in AgCu nanoparticles is not conducive to the generation of optimal active sites for interaction with pathogens. Copper may oxidize more readily to form CuO on the surface, which may alter antifungal activity compared to pure silver nanoparticles. Interactions between metals can cause one metal to suppress the activity of another [38,39]. However, in a study by Medina et al. [40], AgCu showed better antimicrobial activity than monometallic nanoparticles. It was pointed out that the crucial element influencing the effectiveness of a given method is the difference in the methods of experiments. The article by Medina et al. presents the results of a study on the antibacterial properties of nanocomposites based on titanium (TiO_2_), however, which were modified with gold (Au), silver (Ag), copper (Cu), and their complexes (Ag-Cu). The article states that Ag-Cu complexes had the lowest minimum bactericidal concentration (MBC). In our study, we obtained the same results with those complexes. Other in vitro studies, however, confirmed that the addition of Ag and Cu NPs to cosmetic cow udder care products synergizes the effects of these mixtures, which also provides an effective method of *mastitis* prevention, further indicating the potential and new opportunities to discover synergistic biocidal properties of NPs [41].

For bacterial infections, bimetallic nanoparticles (in our case in complexes) show stronger antibacterial properties compared to single nanoparticles, which was also shown in a literature review by Daimari et al. [42]. The research cited by the authors also showed that nanoparticles in complexes have strong activity against antibiotic-resistant bacteria and exhibit anti-inflammatory and antibacterial properties against pathogens that cause, for example, skin diseases or pneumonia.

An article by Madkhali et al. [43] discusses the impact of Fe_3_O_4_ NPs (iron oxide nanoparticles) on reducing biofilm formation by *Candida* strains, especially compared to traditional fluconazole treatment. Our study did not include testing against biofilms, but it is significant that the results of efficacy against fungi varied depending on the concentration of FeCNPs. According to the paper, different concentrations of Fe_3_O_4_ NPs affected fungal resistance, which corresponds with our results, in which FeCNPs showed a significant relationship between concentration and efficacy. In our study, we observed that efficacy was lower at higher concentrations, although none of the concentrations studied showed total fungal death. In contrast, lower concentrations appeared to be more effective against pathogens, which may suggest that too-high concentrations may unnecessarily decrease the activity of nanoparticles.

According to our observations, PtNPs showed the worst results in our antifungal tests. All remaining nanoparticles showed better results in reducing the survival rate of fungi. PtNPs had no significant effect on fungal survival, even at the highest concentrations, which is in line with the results described in the article by Chlumsky et al. [44], in which they were also found to be less effective in biofilm inhibition. In that article, Chlumsky et al. also studied PdNPs, which instead showed 80% inhibition of the growth of planktonic cells and biofilm, achieving 80% CFU reduction at a concentration of 44.5 mg/L, making them more promising candidates for antifungal and antibacterial applications.

Several investigations have explored how nanoparticles affect fungal strains from mastitis-affected cows. While some studies have found that AgNPs and CuNPs have strong antifungal activity, others, like PtNPs, have shown limited efficacy, particularly in biofilm inhibition. This underscores the need for additional in vivo safety testing of such compounds in the field of animal health.

Another important matter is the physicochemical characteristics of NPs, which affect their biocidal properties. PdI is an indicator that can indicate the homogeneity of particles in solution and determines monodispersity when its value is less than 0.1. This parameter can also signal the aggregation of nanoparticles [45]. In the presented research, all samples were characterized by a higher PdI value, meaning that the hydrocolloids were not homogeneous (Table 3). However, the most similar to monodisperse samples were AgFeC and AuCu, reaching PdI values of 0.3, while values indicating the greatest polydispersity were present in FeC (iron NPs coated with carbon) and CuFeC samples (PdI = 0.8). All analyzed nanoparticles exhibited varying sizes of nanoparticles. The hydrodynamic diameter of all types of nanoparticles exceeded 100 nm; however, in the TEM analysis (Figure 1), individual nanoparticles showed small sizes but clustered to form agglomerates. In zeta potential measurements (Table 3), despite a hydrodynamic diameter of more than 100 nm, AgNPs and Ag complexes (AgAu, AgPt, AgFeC) had values around ±30 mV, which is considered the limit of colloidal stability and points to monodisperse rosins without aggregates. Nanoparticles characterized by a zeta potential of ±20 mV have short-term stability, while those with zeta potentials < ±5 mV aggregate quickly [46]. In our research, only Cu had a zeta potential of less than −5 mV. Considering the results obtained, all types of nanoparticles were able to form agglomerates, even if they showed a zeta potential of more than ±30 mV, due to the high PdI values as well as the large size of the hydrodynamic diameter, especially since, when visualized by TEM, individual nanoparticles showed small sizes.

Regarding the antifungal properties of metal nanoparticles, the effect they exhibit is influenced by many factors, such as shape, size distribution, surface chemistry, and agglomeration. It is recognized that smaller nanoparticles have a higher surface-to-volume ratio, so they exhibit better antifungal properties [47]. One of the mechanisms by which nanoparticles interact with microbial cells is the release of ions that can contribute to generating reactive oxygen species and thus damage cells [48]. In addition to this, the antifungal action of metal nanoparticles involves changes in the cell wall, causing surface changes and the formation of pits and deformations, as well as penetration into cells and interaction with internal structures [49]. In the presented study, metal nanoparticles, due to the formation of agglomerates, probably did not penetrate the fungal cells, but they contributed to their destruction in another way—by interacting with the cell wall or secreting toxic ions, which should be investigated in future studies providing additional information about the up-and-coming antifungal solution of metal nanoparticles and their composites.

## 4. Materials and Methods

### 4.1. Biological Material and the Isolated Fungi Cultures

Quarter milk samples were taken from 20 herds containing dairy cattle of the Polish Holstein-Friesian breed. The farms, which kept 10 to 60 milking cows, were located in the Mazowieckie and Podlaskie provinces. Samples were collected between September 2023 and April 2024 from cows that had subclinical or clinical forms of mastitis present in at least one teat (SCC above 200,000/mL). After disinfecting the teats with 70% ethanol for 30 s, milk samples were collected in sterile tubes (n = 67), and microbiological cultures were then performed. Specialized media dedicated to fungal cultures were used, including Sabouraud Chloramphenicol Agar (Bio-Rad, Hercules, CA, USA), Chromogenic Candida Lab-Agar, and DRBC Lab-Agar (Biomaxima, Lublin, Poland). The prepared cultures were incubated at 37 °C for 24–48 h. The colonies that were grown were then identified using a MALD-TOF MS apparatus (Bruker, Poznan, Poland).

### 4.2. Nanoparticles

The nanoparticles used in the experiment were purchased from a commercially available source. The silver, gold, copper, and platinum nanoparticles were purchased from Nano-tech (Warsaw, Poland), while the iron nanoparticles coated with carbon were purchased from 3D-nano (Krakow, Poland).

### 4.3. Determination of the Nanoparticles’ Morphology

The morphology of the purchased nanoparticles was determined using images taken with a transmission electron microscope (TEM, JEM-1220EX, JEOL, Tokyo, Japan).

A hydrocolloid containing the nanoparticles and distilled water was prepared before the images were taken. The prepared mixture was then sonicated (for 2 min), and then the prepared hydrocolloids were placed on molding TEM grids (Formvar on 3 mm 200 mesh Cu grids, Agar Scientific, Stansted, UK). The Formvar grids were then allowed to dry for 24 h at room temperature, after which the images were taken by applying a voltage of 80 keV to the prepared grids.

### 4.4. Determination of the Physicochemical Properties of the Nanoparticles

To determine the zeta potential and size distribution of the nanoparticles, hydrocolloids were prepared by mixing nanoparticles and distilled water to obtain a concentration of 10 ppm. Then, a Zetasizer Nano ZS apparatus (ZEN3500, Malvern Instruments, Malvern, UK) was used to perform the analyses. Dynamic light scattering (DLS) was used to determine the hydrodynamic magnitude.

### 4.5. Laser Emission Spectroscopy Studies of the Nanoparticles

Prior to laser emission spectroscopy studies, dispersed aqueous suspensions containing Ag, Au, Cu, Pt, and FeC (iron NPs coated with carbon) NPs and nanocomplexes of AgAu, AgCu, AgPt, AgFeC, AuCu, AuPt, AuFeC, CuPt, CuFeC, and PtFeC were spotted with a disposable pipette onto 2-inch-diameter Dummy CZ-Si silicon wafers (Microchemicals, Ulm, Germany) and vacuum dried at 50 °C (BMT Vacucell 22L, Brno-Zábrdovice, Czech Republic). The process was repeated 10 times.

Laser emission spectroscopy was carried out in the experimental setup according to Nasiłowska et al. [50]. The plasma was generated using a pulsed Nd:YAG laser, Brio model, by Quantel, wavelength 1064 nm, accumulation (shots number) 1, pulse duration 4 ns, pulse energy 46 mJ, gate delay 500 ns, gate width 500 ns. The laser beam was incident perpendicular to the surface of the sample. The radiation emitted by the plasma was recorded with a spectrometer using an optical head and optical fiber. For each suspension tested, 3–4 measurements were made by moving the sample surface along the x-axis by 1 mm.

### 4.6. Elemental Composition Tests of Nanoparticles (EDX—Energy-Dispersive X-Ray)

Elemental composition studies were performed using an energy-dispersive X-ray (EDX) detector coupled to FEI’s Quanta 250 FEG microscope, according to the methodology of Zielińska-Górska et al. [51].

### 4.7. Determination of Fungal Viability After Treatment with Nanoparticles and Their Complexes

Suspensions were prepared from the isolated fungi. The cultured fungi were dissolved in a solution of sterile NaCl solution. The prepared suspensions had a value of 1.0 McF. The control group was a mixture of 50 µL of sterile water and 50 µL of fungal suspension. Nanoparticle complexes were created by combining two types of nanoparticles in a ratio of 1:1. This mixture was then vortexed. The experimental groups contained 50 µL of fungal suspension, to which 50 µL of nanoparticles was added at different concentrations: 1.56, 3.125, 6.25, 12.5, and 25 mg/L for Au, Ag, Cu, and FeCNPs (iron NPs coated with carbon) and 0.625, 1.25, 2.5, 5, and 10 mg/L for PtNPs. The prepared suspensions and nanoparticle concentrations were pipetted into 96-well plates so that each well contained 100 µL of the mixture. The prepared plates were protected with parafilm to reduce evaporation of the suspensions and were then placed in an incubator for 24 h at a temperature of 37 °C. After this time, 20 µL of XTT Cell Proliferation Assay Kit reagent (Merck, Darmstadt, Germany) was added to each well. The plate was again sealed with parafilm and placed in an incubator for another 24 h. The results were read using spectrophotometric absorbance at a measurement wavelength of 450 nm and a reference wavelength of 690 nm (Elisa reader Infinite M200, Tecan, Durham, NC, USA). The control group had a fungal survival rate of 100%, to which the survival rates in the experimental groups were compared. Numerical criteria were estimated, according to which the biocidal properties of NPs were evaluated as follows: excellent: viability 0–3%; very strong: viability 4–15%; strong: viability 16–30%; good: viability 31–50%; moderate: 51–78%; weak: viability 80–99%; inactive: viability > 100%. The data were analyzed using one-way analysis of variance (ANOVA) in GraphPad Prism 9 software (version 9.2.0, San Diego, CA, USA). Differences at *p* ≤ 0.05 were considered statistically significant.

### 4.8. Preparation of Fungus Suspensions for Additional Experimental Analysis

The previously identified fungal strains were screened on specialized selective media: Yeast Extract Peptone Dextrose Agar (YPD) (Merck, Darmstadt, Germany), Dichloran-rose Bengal Chloramphenicol Agar (DRBC), Candida Ident Agar (Biomaxima, Lublin), and Sabouraud Dextrose Agar (Bio-Rad, Hercules, CA, USA). The strains were cultured on the media for 24 h at 37 °C and then suspended in a 0.9% solution of sterile NaCl, resulting in a suspension density equal to 0.5 on the McFarland scale (OD = 0.5).

### 4.9. Determination of the Minimum Inhibitory Concentration (MIC)

The determination of the minimum inhibitory concentration (MIC) was conducted on a 24-well plate in the presence of Glucose Chloramphenicol Broth (GCB) (Pol-Aura, Warsaw, Poland). Serial dilutions of the AgNPs, AuNPs, CuNPs, PtNPs, FeCNPs (iron NPs coated with carbon), AgCuNPs, AgAuNPs, and AuCuNPs were performed on the plates using concentrations of 1.56, 3.125, 6.25, 12.5, and 25 mg/L, while 0.625, 1.25, 2.5, 5, and 10 mg/L concentrations were used for PtNPs. The pathogen suspension preparation was carried out in accordance with Section 4.8 of this paper prior to the process. Previously prepared fungal suspensions were added to each well after the serial dilutions were conducted. Each plate included a positive control well (without nanoparticles) and a negative control well (without a pathogen or nanoparticles). The plates were then incubated for 24 h at a temperature of 37 °C. After the incubation period, the results were analyzed by determining the presence or absence of turbidity in the wells at the respective concentrations. The absence of turbidity at a given concentration indicated the MIC.

### 4.10. Determination of the Minimum Fungicidal Concentration (MFC)

The pathogen suspension preparation was carried out in accordance with Section 4.8 of this paper prior to the process. The day following the MIC determination, an experiment was conducted to determine the minimum fungicidal concentration (MFC). After incubating the MIC plates for 24 h, a portion of the solution was taken from the wells with a sterile inoculating loop. The sample was then transferred to plates containing the solid media listed in previous sections of the methodology. These plates were incubated for 24 h at 37 °C. After this time, the results were read by taking pictures of the plates using an Interscience Scan 4000 (Interscience, Saint-Nom-la-Bretêche, France), and the MFC was determined by observing pathogen growth. The absence of growth on the plate indicated the minimum biocidal concentration of the nanoparticles used.

### 4.11. Evaluation of the Susceptibility to Antifungal Agents Using the Disc Diffusion Method

The pathogen suspension preparation was carried out in accordance with Section 4.8 of this paper prior to the process. The pathogen suspensions were carpet-seeded. A 100 µL fungal suspension was sampled and distributed equally over the entire surface of the Petri dish, which contained the solid media listed in previous sections of the methodology, using the swab rotation technique. Antifungal discs were applied directly to the dishes with previously inoculated pathogens using the spread plate method. The time between the inoculation of the pathogen suspension and the application of the discs did not exceed 30 min. Discs containing the following antifungal substances were applied to the plates: fluconazole, 10 g/L (FCN10); fluconazole, 25 g/L (FCN25); econazole, 10 g/L (ECN10); ketoconazole, 10 g/L (KCA10); amphotericin, B 20 g/L (AMB20); flucytosine, 1 g/L (FY1); clotrimazole, 10 g/L (CTM10); and nystatin, 100 g/L (NY100) (MASTDISCAST^®^, MAST GROUP, Bootle, UK). The discs were applied using sterilized tweezers; both antifungal discs and blank discs were applied to each dish. The blank discs were moistened each time with 15 µL of 0.9% (9 g/L) NaCl solution as a control. The plates prepared in this way were incubated in an inverted position for 24 h at 37 °C. After this time, the results were evaluated by measuring the zone of inhibition for the fungal growth in millimeters.

## 5. Conclusions

The resistance of pathogens to antimicrobial agents is increasing and is a major problem in the treatment of basic infections in animals and in humans, to whom resistant pathogens spread. This is now a global problem that scientists around the world are working to solve. Among the isolated strains, *C. glabrata* was the most resistant to NPs, but the survival rate of this strain as well as the other strains after NP treatment indicated their high sensitivity to NPs. The nanoparticles showed a synergistic effect, indicating that combining them provides increased biocidal properties. The biocidal properties of NPs varied depending on the concentration and type of NPs. Nevertheless, Ag, Au, Cu, and their complexes showed biocidal properties; in contrast, FeC and PtNPs showed no or weak effects. Only *Diutina* spp. was susceptible to FeCNPs, with a survival rate of 37–53% after application of the highest concentration. PtNPs showed no biocidal properties. All complexes showed very strong biocidal properties, especially AgCuNPs. This complex demonstrated significant antifungal activity, reducing the survival of some strains by up to 90% even at low concentrations (1.5 mg/L) suggesting that this complex should be included in further in vivo studies.

Despite the promising biocidal properties of NPs and their high potential for the prevention and management of *mastitis*, several key issues need to be addressed. It should be kept in mind that nanotechnology and the use of NPs is still a developing subject; therefore, the effects of the long-term use of NPs on livestock health are unknown, and this issue thus requires further research. In the context of their use in practice, it is necessary to consider the type of nanoparticles as well as their origin, methods of synthesis, and physicochemical properties, which affect not only their biocidal properties but also their safety of application. The results of the conducted studies indicate that NPs may be an effective new alternative in the prevention of fungal *mastitis*, which in turn could lead to an increase in milk yields and improve the quality of dairy products and animal welfare. The use of nanoparticles in the prevention of *mastitis* has the potential to modernize breeding practices in the future. Regarding the potential for nanoparticles to be used in the future for the direct prevention of mastitis, it is necessary to conduct thorough studies on their safety in the context of contact with cells. Cell assays will make it possible to determine whether formulations containing nanoparticles interacting with the tissues of a living organism are safe and whether their use leads to unwanted side effects, such as inflammatory reactions or toxicity. However, before nanoparticles reach direct applications, their use in inflammation prevention may be based on surface applications, where their interaction with living cells does not occur. The nanocomplexes could, for example, be used to decontaminate milking equipment or other tools that have come into direct contact with pathogens from the milk of cows suffering from *mastitis*. However, implementing the use of NPs in practice requires not only confirming their in vivo efficacy and safety but also adapting them for use in practice. Monitoring possible side effects in dairy production will also be an important aspect.

## Figures and Tables

**Table 1:** A summary of isolated strains from collected quarter milk samples.

Sample	N	Presence [%]
no growth	22	32.84
contamination	4	5.97
mixed	1	1.49
one strain	0	0
yeasts and fungi	14	20.89
bacteria	24	35.82
algae	2	2.99

**Table 2:** Identified fungal strains.

Species	Score	NCBI Number
*Candida albicans*	2.11	5476
*Candida glabrata*	2.08	5478
*Candida parapsilosis*	2.24	5480
*Diutina rugosa*	2.14	5481
*Diutina rugosa* var. *rugosa*	2.22	5481
*Diutina catenulata*	2.16	45,537

**Table 3:** Zeta potential measurement values, size distribution, and polydispersity index (PdI) for the nanoparticles.

Nanoparticles	Average Hydrodynamic Diameter (nm)		Zeta Potential (mV)		PdI	
	Average	Standard Deviation	Average	Standard Deviation	Average	Standard Deviation
Ag	201.6	4.3	−35.2	1.7	0.4	0.0
Au	295.9	96.2	−14.8	1.2	0.5	0.1
Cu	652.1	174.0	−3.5	0.5	0.4	0.3
Pt	310.2	162.0	−15.3	7.1	0.6	0.2
FeC	746.9	290.0	−27.8	0.9	0.8	0.8
AgAu	308.3	139.2	−30.0	2.5	0.6	0.2
AgCu	378	45.6	−19.7	8.1	0.7	0.0
AgPt	106.1	28.7	−38.1	3.6	0.4	0.1
AgFeC	216.3	3.0	−35.7	1.0	0.3	0.1
AuCu	119.7	7.0	−23.4	1.4	0.3	0.0
AuPt	356.5	43.7	−25.3	3.4	0.7	0.0
AuFeC	165	14.00	−18.0	2.0	0.3	0.0
CuPt	158.8	52.4	−16.8	6.2	0.4	0.1
CuFeC	743.7	373.9	−21	4.5	0.8	0.2
PtFeC	399	136.8	−20.6	0.4	0.5	0.1

**Table 4:** EDX analysis of tested nanoparticle.

Element		Mean Weight Value [%]	Mean Atomic Value [%]
AgAu	Ag	91.45 ± 2.06	95.31 ± 1.01
	Au	8.57 ± 2.07	4.87 ± 1.22
AgCu	Ag	85.08 ± 2.05	77.09 ± 2.86
	Cu	14.92 ± 2.05	22.92 ± 2.86
AgFeC	Ag	16.69 ± 0.71	4.05 ± 1.01
	Fe	48.13 ± 11.03	23.41 ± 10.22
	C	35.20 ± 11.53	71.81 ± 11.38
AgPt	Ag	6.49 ± 1.62	11.13 ± 2.62
	Pt	93.51 ± 1.62	88.99 ± 2.40
AuCu	Au	22.37 ± 5.30	8.60 ± 2.32
	Cu	77.63 ± 5.30	91.40 ± 2.32
AuFeC	Au	1.69 ± 1.02	0.43 ± 0.26
	Fe	11.17 ± 7.83	2.79 ± 2.20
	C	87.43 ± 8.87	97.11 ± 2.28
AuPt	Au	88.55 ± 1.94	88.45 ± 1.93
	Pt	11.61 ± 1.96	11.52 ± 1.97
CuFeC	Cu	2.94 ± 2.50	0.79 ± 0.69
	Fe	32.32 ± 0.88	9.64 ± 0.62
	C	64.64 ± 3.27	89.53 ± 1.28
CuPt	Cu	1.74 ± 0.98	4.48 ± 1.76
	Pt	98.49 ± 0.61	95.52 ± 1.76
PtFeC	Pt	2.69 ± 1.37	0.43 ± 0.33
	Fe	8.30 ± 5.36	2.20 ± 1.27
	C	89.20 ± 6.82	97.91 ± 1.32
Ag		100 ± 0.0	100 ± 0.0
Au		100 ± 0.0	100 ± 0.0
Cu		100 ± 0.0	100 ± 0.0
FeC	Fe	26.91 ± 4.65	7.37 ± 1.60
	C	73.09 ± 4.65	92.63 ± 1.60
Pt		100 ± 0.0	100 ± 0.0

**Table 5:** Minimum inhibitory concentration (MIC) and minimum fungicidal concentration (MFC) evaluated for six isolated fungal strains.

Pathogen Species	NP Type	MIC (mg/L)	MFC
*Diutina rugosa* var. *rugosa*	Ag	6.25	12.5
	Cu	12.5	12.5
	AgAu	6.25	25
	AgCu	25	25
*Candida parapsilosis*	Ag	3.125	12.5
	AgAu	6.25	25
	AgCu	12.5	25
*Candida albicans*	Ag	12.5	12.5
	AgAu	12.5	25
	AgCu	25	25
*Candida glabrata*	Ag	12.5	12.5
	AgAu	12.5	25
	AgCu	25	25
*Diutina catenulata*	Ag	3.125	12.5
	Au	12.5	25
	Cu	25	-
	Fe	12.5	-
	AgAu	6.25	12.5
	AgCu	0.75	12.5
	AuCu	3.125	3.125
*Diutina rugosa*	Ag	6.25	12.5
	AgCu	25	25

**Table 6:** Zones of inhibition for pathogen growth after the application of commercially available discs with antifungal agents. Applied agents: FCN25—fluconazole, 25 g/L; FCN10—fluconazole, 10 g/L; ECN10—econazole, 10 g/L; KCA10—ketoconazole, 10 g/L; AMB20—amphotericin B, 20 g/L; FY1—flucytosine, 1 g/L; CTM10—clotrimazole, 10 g/L; and NY100—nystatin, 100 g/L.

Pathogen Species	Applied Agent	Concentration of the Mycotic (g/L)	Zones of Inhibition (mm; Diameter)
*Diutina rugosa* var. *rugosa*	fluconazole	25	12
	fluconazole	10	8
	econazole	10	14
	ketoconazole	10	20
	amphotericin B	20	6
	flucytosine	1	10
	clotrimazole	10	22
	nystatin	100	8
*Candida parapsilosis*	fluconazole	25	18
	fluconazole	10	0
	econazole	10	15
	ketoconazole	10	40
	amphotericin B	20	6
	flucytosine	1	16
	clotrimazole	10	34
	nystatin	100	8
*Candida albicans*	fluconazole	25	22
	fluconazole	10	20
	econazole	10	14
	ketoconazole	10	22
	amphotericin B	20	10
	flucytosine	1	12
	clotrimazole	10	14
	nystatin	100	14
*Candida glabrata*	fluconazole	25	18
	fluconazole	10	8
	econazole	10	16
	ketoconazole	10	14
	amphotericin B	20	10
	flucytosine	1	32
	clotrimazole	10	8
	nystatin	100	18
*Diutina catenulata*	fluconazole	25	28
	fluconazole	10	0
	econazole	10	20
	ketoconazole	10	30
	amphotericin B	20	10
	flucytosine	1	20
	clotrimazole	10	22
	nystatin	100	16
*Diutina rugosa*	fluconazole	25	28
	fluconazole	10	0
	econazole	10	20
	ketoconazole	10	30
	amphotericin B	20	10
	flucytosine	1	20
	clotrimazole	10	32
	nystatin	100	16

## Data Availability

The data presented in this study are available upon request from the corresponding author due to (specify the reason for the restriction).

## Supplementary Materials

The supporting information can be downloaded at: https://www.mdpi.com/article/10.3390/molecules30102086/s1. Supplementary Figure S1: The zeta potential of the nanoparticles. (A)—Ag, (B)—Au, (C)—Cu, (D)—FeC, (E)—Pt, (F)—AgCu, (G)—AuAg, (H)—AuCu, (I)—AgPt, (J)—AuPt, (K)—CuPt, (L)—FeCAg, (M)—FeCAu, (N)—FeCCu, (O)—FeCPt.

## Abbreviations

The following abbreviations are used in this manuscript:

NPs	nanoparticles
AgNPs	silver nanoparticles
AuNPs	gold nanoparticles
CuNPs	copper nanoparticles
FeCNPs	iron nanoparticles with hydrophilic carbon coating
PtNPs	platinum nanoparticles
AgCuNPs	silver–copper nanoparticle complex
AgAuNPs	silver–gold nanoparticle complex
AuCuNPs	gold–copper nanoparticle complex
AgPtNPs	silver–platinum nanoparticle complex
AgFeCNPs	silver–iron nanoparticle complex
AuPtNPs	gold–platinum nanoparticle complex
AuFeCNPs	gold–iron nanoparticle complex
CuFeCNPs	copper–iron nanoparticle complex
MIC	minimal inhibitory concentration
MFC	minimal fungicidal concentration
FCN10	fluconazole10 g/L
FCN25	fluconazole 25 g/L
ECN10	econazole 10 g/L
KCA10	ketoconazole 10 g/L
AMB20	amphotericin B 20 g/L
FY1	flucytosine 1 g/L
CTM10	clotrimazole 10 g/L
NY100	nystatin 100 g/L
