# Supplementary material for: Nanoparticles as New Antifungals in the Prevention of Bovine Mycotic Mastitis Caused by Candida spp. and Diutina spp.—In Vitro Studies
Source: Molecules. 2025 May 8;30(10):2086. doi: 10.3390/molecules30102086 (PMC12113736; doi:10.3390/molecules30102086)
Supplement: Supplementary file 1 [file molecules-30-02086-s001.zip › molecules-3570309 supplementary.pdf]

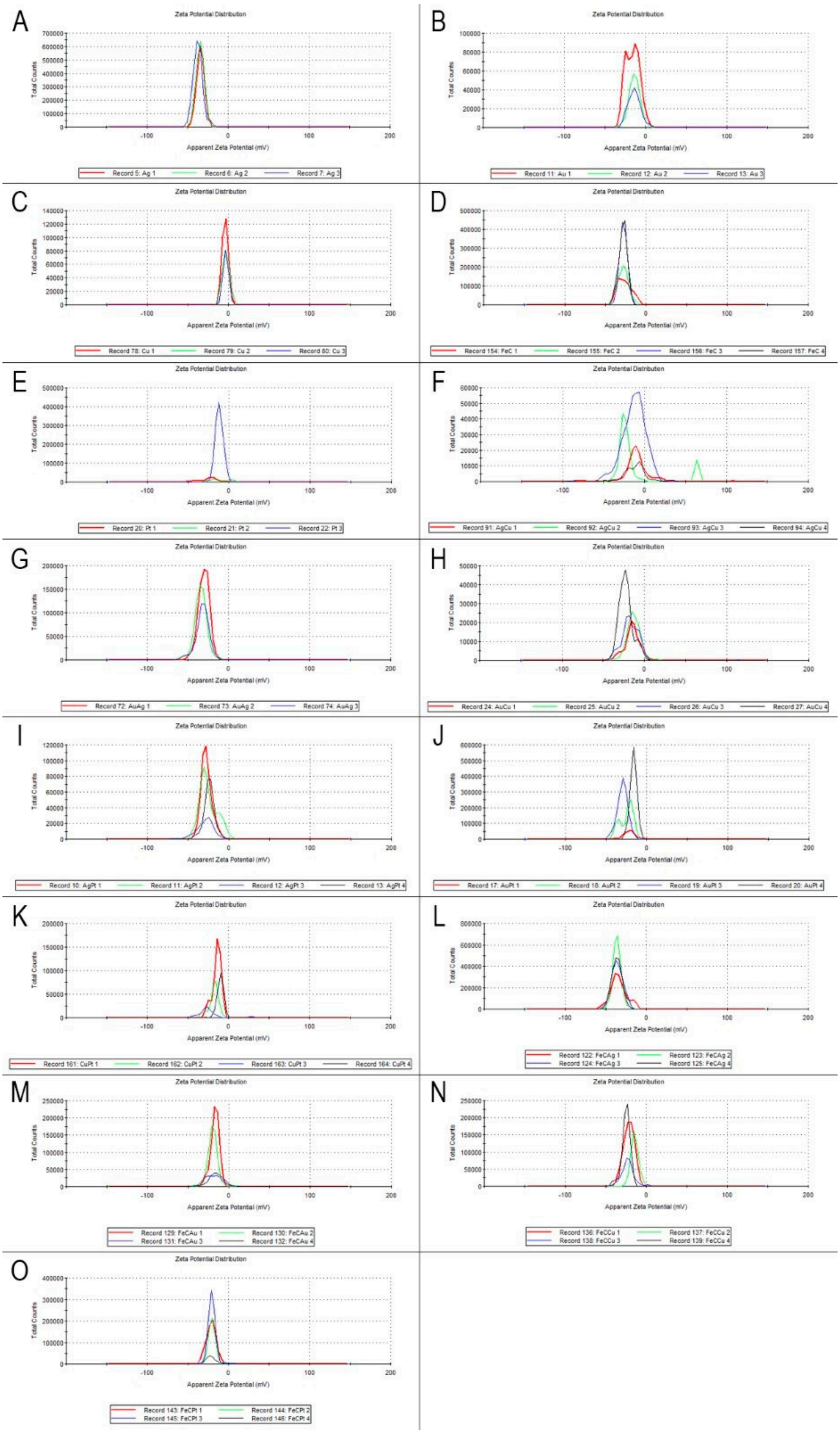

A
Zeta Potential Distribution
Total Counts
Apparent Zeta Potential (mV)
Record 5: Ag 1
Record 6: Ag 2
Record 7: Ag 3
B
Zeta Potential Distribution
Record 11: Au 1
Record 12: Au 2
Record 13: Au 3
C
Zeta Potential Distribution
Record 78: Cu 1
Record 79: Cu 2
Record 80: Cu 3
D
Zeta Potential Distribution
Record 154: FeC 1
Record 155: FeC 2
Record 156: FeC 3
Record 157: FeC 4
E
Zeta Potential Distribution
Record 20: Pt 1
Record 21: Pt 2
Record 22: Pt 3
F
Zeta Potential Distribution
Record 91: AgCu 1
Record 92: AgCu 2
Record 93: AgCu 3
Record 94: AgCu 4
G
Zeta Potential Distribution
Record 72: AuAg 1
Record 73: AuAg 2
Record 74: AuAg 3
H
Zeta Potential Distribution
Record 24: AuCu 1
Record 25: AuCu 2
Record 26: AuCu 3
Record 27: AuCu 4
I
Zeta Potential Distribution
Record 10: AgPt 1
Record 11: AgPt 2
Record 12: AgPt 3
Record 13: AgPt 4
J
Zeta Potential Distribution
Record 17: AuPt 1
Record 18: AuPt 2
Record 19: AuPt 3
Record 20: AuPt 4
K
Zeta Potential Distribution
Record 161: CuPt 1
Record 162: CuPt 2
Record 163: CuPt 3
Record 164: CuPt 4
L
Zeta Potential Distribution
Record 122: FeCAg 1
Record 123: FeCAg 2
Record 124: FeCAg 3
Record 125: FeCAg 4
M
Zeta Potential Distribution
Record 129: FeCAu 1
Record 130: FeCAu 2
Record 131: FeCAu 3
Record 132: FeCAu 4
N
Zeta Potential Distribution
Record 136: FeCCu 1
Record 137: FeCCu 2
Record 138: FeCCu 3
Record 139: FeCCu 4
O
Zeta Potential Distribution
Record 143: FeCPt 1
Record 144: FeCPt 2
Record 145: FeCPt 3
Record 146: FeCPt 4

Supplementary Figure S1. The zeta potential of the nanoparticles. A - Ag, B - Au, C - Cu, D - FeC, E - Pt, F - AgCu, G - AuAg, H - AuCu, I - AgPt, J - AuPt, K - CuPt, L - FeCAg, M - FeCAu, N - FeCCu, O - FeCPt.
